# Spatial spread of local field potential is band-pass in the primary visual cortex

**DOI:** 10.1152/jn.00443.2016

**Published:** 2016-08-03

**Authors:** Agrita Dubey, Supratim Ray

**Affiliations:** Centre for Neuroscience, Indian Institute of Science, Bangalore, India

**Keywords:** primary visual cortex, V1, local field potential, high-gamma, LFP spread

## Abstract

Local field potential (LFP) is a valuable tool in understanding brain function and in brain machine-interfacing applications. However, there is no consensus on the spatial extent of the cortex that contributes to the LFP (its “spatial spread”), with different studies reporting values between a few hundred micrometers and several millimeters. Furthermore, the dependency of the spatial spread on frequency, which could reflect properties of the network architecture and extracellular medium, is not well studied, with theory and models predicting either “all-pass” (frequency-independent) or “low-pass” behavior. Surprisingly, we found the LFP spread to be “band-pass” in the primate primary visual cortex, with the greatest spread in the high-gamma range (60–150 Hz). This was accompanied by an increase in phase coherency across neighboring sites in the same frequency range, consistent with the findings of a recent model that reconciles previous studies by suggesting that spatial spread depends on neuronal correlations.

## Abstract (precis)

*Local field potential (LFP) has become a promising candidate for neural prosthesis, but how different frequencies of the LFP spread has not been studied experimentally, with theoretical models predicting either an “all-pass” (all frequencies spread equally) or “low-pass” (lower frequencies spread farther than higher frequencies) behavior. Our findings suggest that the LFP spread is “band-pass,” with frequencies in the high-gamma (60–150 Hz) range spreading significantly more than both lower (20–40 Hz) and higher (>250 Hz) frequencies*.

## NEW & NOTEWORTHY

*Local field potential (LFP) has become a promising candidate for neural prosthesis, but how different frequencies of the LFP spread has not been studied experimentally, with theoretical models predicting either an “all-pass” (all frequencies spread equally) or “low-pass” (lower frequencies spread farther than higher frequencies) behavior. Our findings suggest that the LFP spread is “band-pass,” with frequencies in the high-gamma (60–150 Hz) range spreading significantly more than both lower (20–40 Hz) and higher (>250 Hz) frequencies*.

in recent years, there has been a resurgent interest in the local field potential (LFP), which is obtained by low-pass filtering the extracellular signal (typically below ∼250 Hz) recorded from a microelectrode and is thought to reflect synaptic inputs, dendritic processing, subthreshold membrane oscillations, and spike after-potentials (Buzsáki et al. 2012; Einevoll et al. 2013; Mitzdorf 1985). LFP indexes synaptic events that are causal to spiking, captures network oscillations that are associated with different behavioral states (Buzsáki 2006; Buzsáki and Draguhn 2004), and is correlated with other measures of brain activity, such as the blood oxygenation level-dependent signal (Goense and Logothetis 2008; Logothetis et al. 2001). Therefore, it provides an easily accessible and unique window for understanding the properties of the neuronal network near the microelectrode. In addition, because it is easily recorded and is more tolerant to microelectrode movements compared with spikes, LFP is also a promising candidate for brain machine-interfacing applications (Andersen et al. 2014).

To gain insights about the neuronal network from LFP, it is important to first estimate the area of the brain tissue around the microelectrode that contributes to this signal, which is referred to as the spatial or cortical spread (processes contributing to the spatial spread are discussed in the first paragraph of results). However, previous studies that have estimated the spatial spread of the LFP have reported widely varying values (Berens et al. 2008; Kajikawa and Schroeder 2011; Katzner et al. 2009; Kreiman et al. 2006; Liu and Newsome 2006; Xing et al. 2009), ranging from a few hundred micrometers (Katzner et al. 2009; Xing et al. 2009) to several millimeters (Kajikawa and Schroeder 2011; Kreiman et al. 2006). Lindén and colleagues (2011) attempted to reconcile these results by showing that the spatial spread depends on the correlation in synaptic activity in the network: when the synaptic activity is uncorrelated across the neural population, spatial spread is extremely circumscribed (a few hundred microns). Otherwise, the spread is determined by the spatial extent of correlated activity.

In the primary visual cortex (V1), the correlation in the network (as measured indirectly using field-field or spike-field coherence) during spontaneous activity, as well as the change in correlation upon stimulus presentation, is frequency dependent, with a greater change in correlation in alpha (8–12 Hz), gamma (30–60 Hz), and high-gamma (60–150 Hz) bands (Jia et al. 2013a; Srinath and Ray 2014). However, whether the spatial spread is frequency dependent in V1 in a manner that depends on the correlation profile has not been studied in detail. Furthermore, the profile of spatial spread as a function of frequency could provide important information about the extracellular medium. For example, some studies have suggested the extracellular medium to be capacitive (Bédard et al. 2004, 2006, 2010), which predicts that lower frequencies should travel farther than high frequencies, yielding a “low-pass” spread vs. frequency profile. Other studies have shown the cortical medium to be purely resistive, which predicts an “all-pass” response, where all frequencies spread equally (Logothetis et al. 2007). Finally, some models have predicted a low-pass spatial spread, even in a purely resistive medium, owing to dendritic filtering effects (Lindén et al. 2010; Pettersen and Einevoll 2008) and a preferential transfer of temporal correlations in presynaptic spike trains to correlations in single-neuron LFPs at lower frequencies (as discussed later) (Einevoll et al. 2013; Łęski et al. 2013).

We estimated the LFP spread as a function of frequency recorded from the V1 of two monkeys. To our surprise, we found the LFP spread to be “band-pass,” with frequencies in the high-gamma range spreading significantly more than both lower and higher frequencies, mirroring an increase in phase coherence across neighboring sites in the same frequency range.

## MATERIALS AND METHODS

### 

#### Behavioral task and recording.

**Fig. 1.**
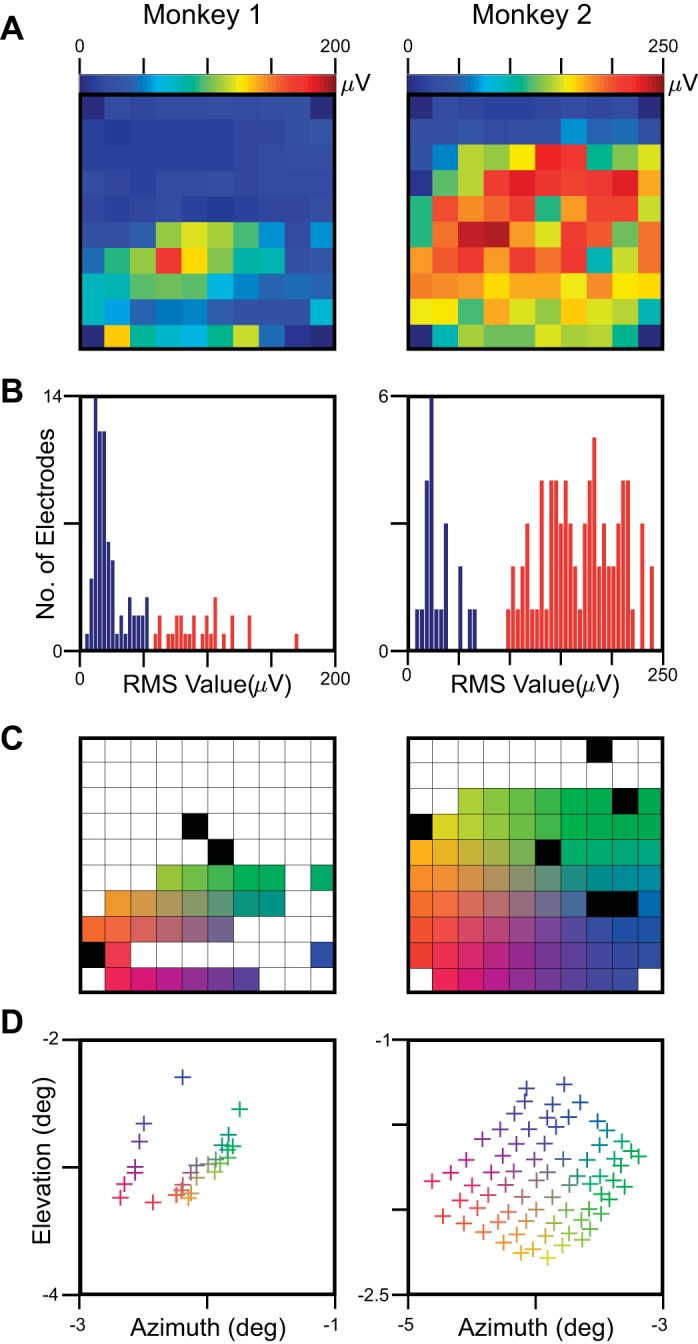
Electrode-selection criteria for LFP. The difference in root mean square (RMS) value between stimulus (40–100 ms) and baseline (−100 to −40 ms) epoch was computed for each of the 9 × 9 or 11 × 11 stimulus positions, and the maximum value across all of these positions was computed. *A*: heat map of the maximum RMS values averaged across all recording sessions for the 96 active microelectrodes in a 10 × 10 microarray grid. *B*: the histogram of the maximum RMS values. The electrodes with maximum RMS value above a particular cutoff (shown in red) were used for further analysis. *C*: selected electrodes, color coded based on their position on the microarray grid. Red/yellow, left edge of the grid and most medial (almost parallel to the midline); yellow/green, top edge, most anterior. The electrodes shown in black had high impedance (>2,500 kΩ) and were excluded. *D*: receptive field (RF) centers of the selected (27 for *Monkey 1* and 71 for *Monkey 2*) electrodes. As expected from the anatomy of V1, the movement in the lateral direction (red to blue or yellow to green) made the RFs more foveal. Similarly, the movement in the anterior direction (red to yellow or blue to green) decreases the elevation.

All of the animal protocols used in this study were approved by the Institutional Animal Care and Use Committee of Harvard Medical School. The recordings were performed on two male rhesus monkeys (*Macaca mulatta*; 11 and 14 kg). Scleral search coil and a head post were implanted into the monkey before training. Once the monkey had learned the behavioral task, a 10 × 10 microelectrode array grid (Blackrock Microsystems, Salt Lake City, UT; 96 active electrodes) was implanted in V1 at the right cerebral hemisphere (∼15 mm from the occipital ridge and 15 mm lateral from the midline). The microelectrodes were 1 mm long, separated by 400 μm, and had an impedance of ∼700 kΩ at 1 kHz. As the total thickness of visual cortex is 2–2.5 mm (Hubel and Wiesel 1977; Wagstyl et al. 2015), the microelectrodes were expected to be in *layer 4* if the cortical thickness was relatively less, the base of the array was completely flush with the cortex (no gap), and the insertion was perpendicular to the cortex; otherwise, they could be in *layer 2/3*. Histology has not been performed to identify the layer. The receptive fields (RFs) estimated from the microelectrode recordings were in the lower-left quadrant of the visual field and centered at 3–5° eccentricity (Fig. 1). Other studies from the same dataset have been reported previously (Ray and Maunsell 2010, 2011a, b; Srinath and Ray 2014).

The monkeys performed an orientation-change detection task, where they fixated within 1° of a small dot (0.05–0.10° diameter) located at the center of a cathode ray tube video display (100 Hz refresh rate, 1,280 × 768 pixels, gamma corrected), while two achromatic odd-symmetric Gabor stimuli were synchronously flashed for 200 ms with an interstimulus period of 300 ms. The monkey was cued to attend to a low-contrast (∼2.6% for *Monkey 1* and ∼5.4% for *Monkey 2*) Gabor stimulus outside of the RF and respond to a change in the orientation of the Gabor stimulus by 90° in one of the presentations by making a saccade within 500 ms of the change. The second stimulus was a small Gabor (sigma of 0.1° for *Monkey 1* and 0.05° for *Monkey 2*) with a spatial frequency of 5 cycles/°, full contrast, and 1 of 4 different orientations: 0°, 45°, 90°, and 135°, flashed in a random order on 1 of 81 (9 × 9 rectangular grid spanning an azimuth of −3.2 to −1.2° and elevation of −4.1 to −2.1°, *Monkey 1*) or 121 (11 × 11 rectangular grid spanning an azimuth of −5.25 to −2.25° and elevation of −3.25 to −0.25°, *Monkey 2*) positions. We collected data in 6 (*Monkey 1*) and 24 (*Monkey 2*) recording sessions. On average, at each location, stimuli were presented 16.9 times (range 11–21) for *Monkey 1* and 13.3 times (range 8–17) for *Monkey 2* (pooled across orientations).

Raw data were band-pass filtered between 0.3 Hz (Butterworth filter, first order, analog) and 500 Hz (Butterworth filter, fourth order, digital) and digitized at 2 kHz (16-bit resolution) to obtain the LFP. Multiunit activity (MUA) was extracted by filtering the raw signal between 250 Hz (Butterworth filter, fourth order, digital) and 7,500 Hz (Butterworth filter, third order, analog), followed by an amplitude threshold (set at ∼6.25 and ∼4.25 of the signal SD for the 2 monkeys). The current source density (CSD), which is the scaled Laplacian of the LFP, was calculated by subtracting the mean of instantaneous voltages of four neighboring electrodes (separated by 400 μm) from the instantaneous voltage of that particular electrode. Specifically, For a two-dimensional (2D) microelectrode array, the CSD at coordinate (*x*,*y*) of a particular electrode is defined as (Chen et al. 2011)

(1) $$CSD\left(x,y\right)=-\frac{4\epsilon }{d^{2}}\left[V\left(x,y\right)-\frac{V\left(x-1,y\right)+V\left(x+1,y\right)+V\left(x,y-1\right)+V\left(x,y+1\right)}{4}\right]$$

For simplicity, we ignored the term $-\frac{4\epsilon }{d^{2}},$ such that the CSD traces also had units of voltage. Note that the negative sign was also ignored, although this did not affect our RF size estimation, as we used root mean square (RMS) values (see RF estimation and electrode selection).

This method for CSD computation uses only differences in the LFP in the lateral direction, implicitly assuming very little variation in the vertical direction. For example, the rich distribution of sources and sinks spreads across cortical layers, as observed in CSDs computed across the vertical direction [see, for example, Fig. 1*C* of Kajikawa and Schroeder (2011)] cannot be captured in the CSDs computed laterally. Some of these issues could potentially be addressed by using microelectrode arrays that record neuronal activity both laterally and vertically from various cortical layers (Csicsvari et al. 2003), such as the Buzsáki 256 probe [Berényi et al. (2014), and see http://neuronexus.com/custom-design/dr-gyorgy-buzsaki], or by using more sophisticated algorithms, such as the inverse CSD that can incorporate more general assumptions regarding the geometrical arrangement of electrode contacts (Łęski et al. 2011). We did not follow this procedure for simplicity and for better comparison with the results of Kajikawa and Schoreder (2011), who also used traditional CSD.

#### RF estimation.

**Fig. 2.**
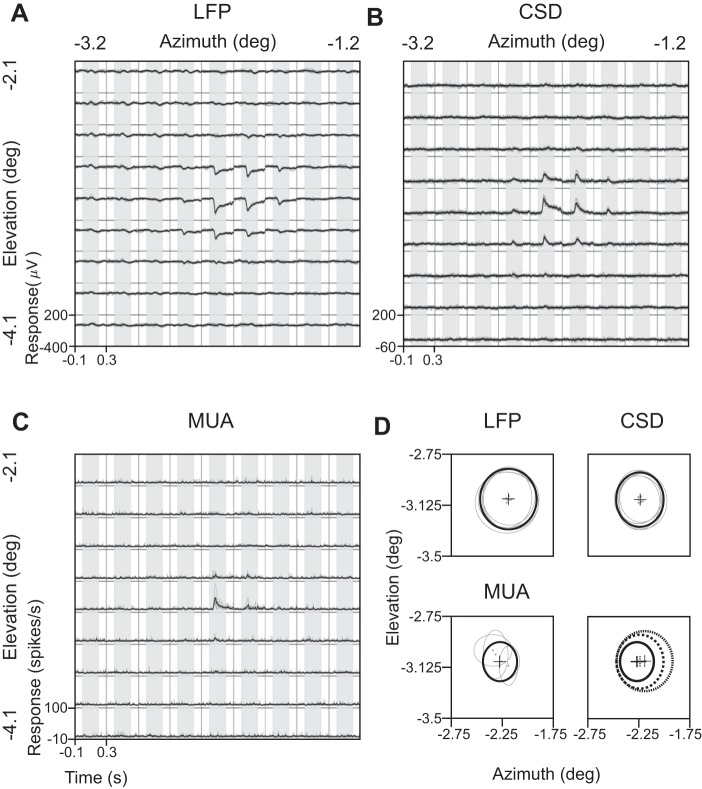
Comparison of LFP, CSD, and MUA spreads from an example electrode recorded from *Monkey 1*. *A–C*: evoked LFP, CSD, and MUA responses, respectively, averaged across trials for 6 recording sessions (shown in gray; not all traces are visible due to a high degree of overlap) at each of the 81 stimulus positions (9 × 9 rectangular grid spanning 2 × 2° of visual space). Mean responses across recording sessions are plotted in black traces. The duration for which stimulus was presented (200 ms) is shown in light gray. *D*: estimated RF sizes and centers for each recording session (gray traces), as well as the mean across sessions (black), estimated by fitting a 2D Gaussian on the data (see text for details). The mean responses (black traces) are replotted in the plot on the *bottom right* in dotted (LFP), dashed (CSD), and solid (MUA) traces for comparison. Note that the estimated RF sizes follow the order: LFP > CSD > MUA.

To estimate the RF sizes, we first averaged the LFP, CSD, and MUA responses across stimulus repeats separately for all of the 9 × 9 (*Monkey 1*) or 11 × 11 (*Monkey 2*) stimulus positions (as shown in Fig. 2). For LFP and CSD, we calculated the RMS values of the mean signal for stimulus (40–100 ms) and baseline (−100 to −40 ms) epochs. This time duration was chosen to capture better the transient negative peak in the LFP and positive peak in CSD; the choice of a different interval, such as 0–100 or 0–200 ms, yielded similar results. RMS values were used because they could be compared directly with the results obtained from the computation of energy; the use of other measures, such as peak negativity [as used by Xing and colleagues (2009)], yielded similar results. We subtracted the RMS values obtained during the baseline period from the stimulus period and fitted the following 2D Gaussian function

(2) $$f\left(x,y\right)=C*\exp\left(-\left(\frac{x_{g}{}^{2}}{2\sigma_{x}^{2}}+\frac{y_{g}{}^{2}}{2\sigma_{y}^{2}}\right)\right)$$

where *C* is the scaling factor, and *σ*_*x*_ and *σ*_*y*_ denote the SD of the Gaussian along major and minor axes. The terms *x*_*g*_ and *y*_*g*_ are defined as

$$\begin{array}{l} x_{g}=(x-x_{o})\;\cos\left(\theta \right)-(y-y_{o})\;\sin\left(\theta \right)\\ y_{g}=(x-x_{o})\;\sin\left(\theta \right)+(y-y_{o})\;\cos\left(\theta \right) \end{array}$$

where θ is the angle of rotation with respect to the coordinate system (*x*_*o*_ and *y*_*o*_) and denotes the center of the 2D Gaussian. We defined the RF center as (*x*_*o*_,*y*_*o*_) and size as $\sqrt{\left(\frac{\sigma_{x}^{2}\;+\;\sigma_{y}^{2}}{2}\right)}.$

For MUA responses, instead of RMS values, we used the difference between the mean firing rates between stimulus and baseline epochs. The use of RMS values for firing rates yielded similar results.

#### Electrode selection.

As in our previous reports, we used only those electrodes for analysis that yielded reliable LFP responses; here, we explain the selection process in detail. First, the difference in RMS value of the LFP between stimulus (40–100 ms) and baseline (−100 to −40 ms) epochs was computed for each of the 9 × 9 or 11 × 11 stimulus positions, and the maximum value across all positions (corresponding to the response when stimulus was near the center of the RF) was used for further analysis. Figure 1*A* shows these values as a heat map, averaged across all recording sessions (6 for *Monkey 1* and 24 for *Monkey 2*) for the 96 active microelectrodes. The entire upper half of the array for *Monkey 1* and upper two rows of *Monkey 2* did not respond to visual stimulation. We did not find any MUA activity in these electrodes either. The reasons are unclear and could possibly be due to improper insertion of the upper side of the array in the cortex (the impedances were in proper range though). We selected electrodes whose maximum RMS value exceeded a particular threshold [shown in the histogram in Fig. 1*B*; we used thresholds of 54 μV (*Monkey 1*) and 95 μV (*Monkey 2*); the use of lower cutoffs increased the number of electrodes, but the RFs were less stable across electrodes]. In ∼5% of the sessions, the estimated RF size was either too small (<0.05°) or too large (>0.5°), or the estimated RF center was beyond the area where the probes were flashed. These sessions were not used for further analysis. The RF centers of the remaining sessions were very stable (SD of <0.1°). Figure 1*C* shows the selected electrodes, color coded on the basis of their position on the grid. The left edge of the grid was most medial (almost parallel to the midline), whereas the top edge was most anterior. Some electrodes had high impedance (>2,500 kΩ) and were ignored for further analysis. The RF centers for the selected electrodes are shown in Fig. 1*D*. As expected from the anatomy of V1, the movement in the lateral direction made the RFs more foveal. Similarly, the movement in the anterior direction decreases the elevation.

To avoid multiple counting of some electrodes, for each electrode, we averaged the waveforms across selected sessions at each position (Fig. 2*A*) and calculated the RF for this pooled data to get a single estimate of RF size and center per electrode (Fig. 2*D*). For CSD analysis, we could only use a subset of these electrodes that had 4 neighboring electrodes at 400 μm, yielding 16 and 39 electrodes for the 2 monkeys (same procedure as LFP). MUA data were less stable across days (Fig. 2*C*), so for a particular electrode, we only used sessions for which at least 10 spikes/s were recorded when the probe was at the center of the RF and only used electrodes for which at least 50% of the sessions were selected. This yielded 18 and 67 electrodes for the 2 monkeys (for these electrodes, 76.8% and 89.9% of the sessions were used). As for LFP and CSD, data were pooled across selected sessions, and a single estimate of RF was computed for each electrode. For all three measures, the final RF centers and sizes computed from the pooled data were very similar to the average of the RF centers and sizes computed for individual sessions.

#### Conversion from visual to cortical spread.

The cortical/spatial spread of LFP (measured in micrometers) was computed from the visual spread (estimated RF size, measured in degrees) using a model proposed by Xing and colleagues (2009). In their model, the visual spread of MUA (*σ*_*vMUA*_) was assumed to be a function of the magnification factor (MF), cortical spread of MUA (*σ*_*cMUA*_), visual spread of single-unit activity (SUA; *σ*_*vSUA*_), and visual variation of SUA (*σ*_*vv*_). Similarly, the visual spread of LFP (*σ*_*vLFP*_) was a function of MF, cortical spread of LFP *σ*_*cLFP*_, *σ*_*vSUA*_, and *σ*_*vv*_. The difference in *σ_vLFP_^2^* and *σ_vMUA_^2^* yielded the cortical/spatial spread of LFP in terms of MF, visual spreads of LFP and MUA, and the cortical spread of MUA

(3) σcLFP=MFσ2vLFP2−MFσ2vMUA2+σcMUA2

where *σ*_*vLFP*_ and *σ*_*vMUA*_ are the estimated RF sizes of LFP and MUA (see *RF estimation* and *Electrode selection*). The spatial spread of MUA (*σ*_*cMUA*_) was set at 60 μm, which was the value used by Xing and colleagues (2009) based on previous studies (Buzsáki 2004; Gray et al. 1995).

We computed MF (in units of millimeters per degree) as the ratio of the cortical distance between the electrode pairs to the visual distance between the estimated RF centers [same way as done by Xing and colleagues (2009)]. MFs for LFP were 2.43 mm/° for *Monkey 1* and 2.71 mm/° for *Monkey 2*. Similarly, MFs were 2.15 mm/° (*Monkey 1*) and 2.30 mm/° (*Monkey 2*) for CSD and 2.75 mm/° (*Monkey 1*) and 2.71 mm/° (*Monkey 2*) for MUA. Although previous studies (Palmer et al. 2012; Van Essen et al. 1984) have reported anisotropy in MF—specifically, larger RF sizes along the horizontal meridian and the smaller along the vertical meridian—we assumed the MF to be similar in both horizontal and vertical directions, because our stimulus spanned only 2° × 2° (*Monkey 1*) or 3° × 3° (*Monkey 2*) in visual space. We used the MF for LFP in *Eq. 3* to compute the cortical spread; results were similar if MF obtained from MUA were used instead.

#### Time-frequency analysis.

Time-frequency analysis was performed using the matching pursuit (MP) (Mallat and Zhang 1993) algorithm. MP is an iterative algorithm that decomposes the signal into a linear combination of waveforms selected from an over-complete dictionary. An over-complete dictionary of Gaussian-modulated sinusoidal functions (Gabor functions) is first created by shifting, scaling, and modulating a single Gabor function (these functions are called “atoms”). In the first iteration, the atom that best matches the signal (i.e., has the largest inner product with it) is chosen from the dictionary, and the atom is approximated to the signal with a coefficient equal to the inner product (i.e., the original signal is considered as a sum of the atom weighted by the inner product and a residue). In the next iteration, residue replaces the signal, and the procedure continues. The time-frequency energy distribution of the atoms selected in the MP decomposition is derived from the Wigner-Ville distribution. Mathematical details of this method are presented elsewhere (Ray et al. 2008b). MP can capture both transient and rhythmic components in the signal, since the over-complete dictionary contains some Gabor atoms that are well localized in the time domain (to capture transients) and others that are well localized in the frequency domain [to capture rhythms, see Chandran KS et al. (2016) for a detailed comparison with other methods].

MP was performed on signals of length 2,048 (−549.5 to 474 ms at 0.5 ms resolution, where 0 denotes the time of stimulus onset), yielding a 2,048 × 2,048 array of time-frequency energy values (with a time resolution of 0.5 ms and frequency resolution of 2,000/2,048 = ∼1 Hz). This was downsampled further by a factor of eight in the time domain and a factor of two in the frequency domain, yielding a time resolution of 4 ms and a frequency resolution of ∼2 Hz.

Time-frequency energy difference plots (see Fig. 5) were obtained using

(4) $$D\left(t,\omega \right)=10\times \left(\log_{10}E\left(t,\omega \right)-\log_{10}B\left(\omega \right)\right)$$

where *E*(*t*,ω) is the mean energy averaged over trials and recording sessions at time *t* and frequency ω obtained from the MP algorithm. *B*(ω) is the mean baseline power averaged across all stimulus locations computed by first averaging the energy in the −100 to 0 ms time interval at each frequency.

#### Coherence analysis.

If the Fourier coefficients of two windowed signals in polar coordinates are denoted as *A*_*k*_(*f*)*e*^*j*ϕ_*k*_^^(*f*)^ and *B*_*k*_(*f*)*e*^*j*θ_*k*_^^(*f*)^, where *f* is the frequency, and *k* = 1, 2, …*n* denotes the trial number, then phase-coherence or phase-locking value is calculated by (Lachaux et al. 1999)

(5) $$C_{phase}(f)=\frac{1}{N}\left|\sum_{k}e^{j\left(\phi_{k}(f)-\theta_{k}(f)\right)}\right|$$

We used Chronux (Bokil et al. 2010), which is an open-source data analysis toolbox, for computing the phase coherence between electrode pairs. This toolbox implements the multitaper method, which uses a Slepian taper (or window) (Mitra and Pesaran 1999; Thomson 1982). We used a single taper to maximize the frequency resolution.

For this analysis, one of the electrodes was considered as the reference, and phase coherence was calculated between this electrode and all of the other electrodes on the grid using trials for which the probe stimulus was closest to the estimated RF center of the reference (such that the reference electrode was always well stimulated). This procedure was repeated for all of the electrodes on the grid, yielding 702 (27 × 26) and 4,970 (71 × 70) pairs. Note that here, each unique pair was counted twice, because stimulus conditions were different depending on which electrode was chosen as the reference (for example, phase coherence between electrodes x and y was counted twice, once using trials for which the probe stimulus was near the center of the RF of electrode x and for the second case, for trials in which the probe stimulus stimulated electrode y). Finally, the electrode pairs were divided into four categories depending on the interelectrode distance (4 pairs for *Monkey 2* had distances >4 mm and were excluded), and the median phase coherence across electrode pairs within each category was computed (see Fig. 7).

#### Bootstrapping.

The standard deviation (SD) was computed using bootstrapping. Given a sample of *n* elements, first, we created a new sample (termed “resample” or bootstrap sample) by randomly taking *n* elements, with replacement, from the original dataset and computing its median. This process was iterated 1,000 times, providing 1,000 medians. The SE was simply the SD of this population of medians.

## RESULTS

The term “spatial spread” can be interpreted in different ways, since different studies have used different experimental techniques or models to estimate it. One strategy is to inject a current in the cortical medium and study how potential falls with distance, irrespective of how this current might excite the neural population (Logothetis et al. 2007). Another strategy is to excite a single neuron (in a model) and study how the extracellular potential generated, due to the transmembrane currents across the neuron, falls with distance (Lindén et al. 2010; Pettersen and Einevoll 2008). Here, the spread is passive, since in the model, the generation of an action potential in a neuron does not lead to the generation of more action potentials in the network. Unfortunately, these approaches cannot be replicated in physiological experiments in which a neuron, once excited, may excite more neurons, thereby generating more active sources and sinks. We therefore use the same strategy as used in previous neurophysiological studies (Kajikawa and Schroeder 2011; Xing et al. 2009), where small parts of cortex near the recording microelectrode are stimulated by the presentation of small stimuli, and the falloff in LFP magnitude, as the stimulated part moves away from the recording electrode, is measured. Following these studies, we call this the spatial spread as well, but we note that the spread measured this way depends also on neural network dynamics and could have contributions from both passive and active processes. As we discuss later, some differences between modeling and experimental results could be due to the differences in the way spatial spread is defined and estimated.

We recorded spike and LFP signals from two male rhesus monkeys using a 10 × 10 array (Blackrock Microsystems) implanted in the V1, while they attended to a Gabor stimulus presented in the opposite hemifield of the RFs of the recorded sites and performed an orientation-change detection task [same task as used in previous studies: Ray and Maunsell (2010, 2011a); Srinath and Ray (2014); see materials and methods for details]. To estimate the spatial spread, we first estimated the RF sizes by flashing a small Gabor stimulus in a random sequence at 81 (*Monkey 1*, 9 × 9 rectangular grid spanning 2 × 2° of visual space) or 121 (*Monkey 2*, 11 × 11 grid spanning 3 × 3° of visual space) locations spanning all of the RFs. Figure 2*A* shows the evoked LFP response recorded from an electrode in *Monkey 1* for six recording sessions, as well as the mean response, in response to probe stimuli flashed at different locations for 200 ms. The evoked LFP responses were very robust across sessions (the gray traces are almost completely overlapping with the black trace and are not visible in most cases). Figure 2, *B* and *C*, shows similar plots for CSD (computed using *Eq. 1*) and MUA. For LFP and CSD, we estimated the RF centers and sizes by first computing the difference in the RMS value between stimulus (40–100 ms poststimulus onset) and baseline (−100 to −40 ms) epochs at each location and then fitting a 2D Gaussian (*Eq. 2*). For MUA, mean firing rates were used instead of RMS values. Note that the MUA plots have more variability across days, because the units comprising the MUA were less tolerant toward small changes in the electrode position. RF sizes and centers for each of the six recording sessions are shown in Fig. 2*D*. We averaged the data across recording sessions for which robust activity was observed (Fig. 2, *A–C*; see materials and methods for details) and computed the RF parameters by fitting a 2D Gaussian to the pooled data. The RF size of LFP (sigma of the fitted 2D Gaussian = 0.21°; *Eq. 2*) was greater than that of CSD (sigma = 0.19°), which was greater than MUA (sigma = 0.14°). These results did not depend appreciably on the choice of metric (RMS vs. peak negativity/positivity) or whether data were normalized before pooling across sessions.

### 

#### Comparison of LFP, CSD, and MUA spreads.

**Fig. 3.**
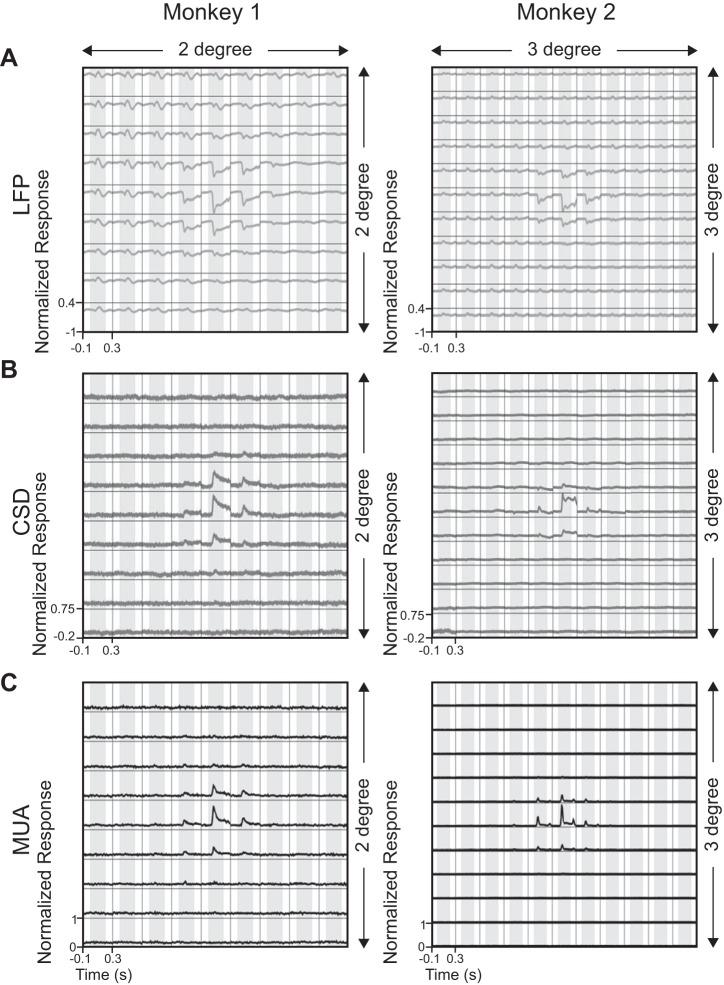
Population responses of LFP, CSD, and MUA for both of the monkeys. *A*: evoked LFP responses averaged over 27 (*Monkey 1*) and 71 (*Monkey 2*) electrodes after first normalizing, shifting, and averaging the traces, such that for each electrode, the maximum response occurred near the center (see text for details). *B*: mean CSD responses across 16 (*Monkey 1*) and 39 (*Monkey 2*) electrodes. *C*: mean MUA responses across 18 (*Monkey 1*) and 67 (*Monkey 2*) electrodes. CSD and MUA traces for individual electrodes were shifted by the same amount as for the LFP traces.

Similar results were obtained for our full dataset of 27 LFP, 16 CSD, and 18 MUA electrodes for *Monkey 1* and 71 LFP, 39 CSD, and 67 MUA electrodes for *Monkey 2* (see materials and methods and Fig. 1 for details of the electrode-selection procedure). For a direct visual comparison, we normalized, centered, and subsequently averaged the LFP, CSD, and MUA signals across all electrodes for *Monkeys 1* and *2* (Fig. 3, *A–C*). Specifically, we first normalized the signals for each electrode by dividing by the absolute maximum response across all stimulus positions. Next, the array of normalized traces (9 × 9 for *Monkey 1* and 11 × 11 for *Monkey 2*) was shifted, such that the position with the largest response was at the center of the grid. This allowed us to average the traces across electrodes (the traces were shifted by the same amount for LFP, CSD, and MUA; note that fewer electrodes contributed to the positions at the edges because of this procedure). This averaging procedure is meaningful, because our microelectrode grid spanned only ∼4 mm^2^ of cortical area, and therefore, the RF sizes were very similar (as described below).

**Fig. 4.**
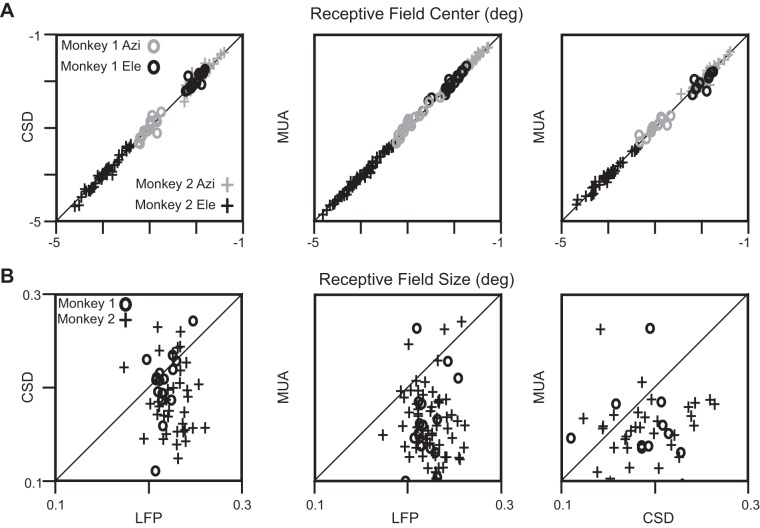
Comparison of RF centers and sizes. *A*: azimuths (Azi; gray) and elevations (Ele; black) of the RF centers for *Monkey 1* (o) and *Monkey 2* (+). The LFP vs. CSD comparison (*left*) is shown for *n* = 16 (*Monkey 1*) and *n* = 39 (*Monkey 2*) electrodes, LFP vs. MUA (*middle*) for *n* = 18 (*Monkey 1*) and *n* = 67 (*Monkey 2*) electrodes, and CSD vs. MUA for *n* = 11 (*Monkey 1*) and *n* = 31 (*Monkey 2*) electrodes. *B*: same plots for RF sizes.

Figure 4, *A* and *B*, shows the comparison of RF centers and sizes between LFP and CSD, LFP and MUA, and CSD and MUA for both the monkeys. RF centers were comparable for all of the three cases (the median difference in azimuth and elevation values across any 2 measures were not significantly different from 0; *P* = 0.57, 0.97; 0.92, 0.61 and 0.45, 1 for *Monkey 1*, and *P* = 0.71, 0.72; 0.52, 0.74 and 0.74, 0.95 for *Monkey 2* for the 3 columns, Kruskal-Wallis test). The RF sizes, however, followed the order LFP > CSD > MUA: the medians and SEs (computed by bootstrapping; see materials and methods for details) of the RF sizes of LFP, CSD, and MUA were 0.23 ± 0.007°, 0.21 ± 0.007°, and 0.16 ± 0.012° for *Monkey 1* and 0.23 ± 0.003°, 0.18 ± 0.009°, and 0.16 ± 0.005° for *Monkey 2*, respectively. Except the LFP vs. CSD comparison for *Monkey 1*, which showed the trend but did not reach significance, the median difference between any two measures was significantly different from zero: LFP vs. CSD, *P* = 0.21, *n* = 16 for *Monkey 1* and *P* = 1.89 × 10^−4^, *n* = 39 for *Monkey 2*; LFP vs. MUA, *P* = 4.45 × 10^−4^, *n* = 18 for *Monkey 1* and *P* = 2.52 × 10^−16^, *n* = 67 for *Monkey 2*; CSD vs. MUA, *P* = 0.04, *n* = 12 for *Monkey 1* and *P* = 4.81 × 10^−4^, *n* = 36 for *Monkey 2*, all comparisons using Kruskal-Wallis test. The use of mean instead of median (using paired *t*-test instead of Kruskal-Wallis test) yielded similar results.

To compute the cortical spread of LFP (measured in micrometers) from RF size (or visual spread, measured in degrees), we used a model proposed by Xing and colleagues (2009) (*Eq. 3*; see materials and methods and their paper for details). This model requires the MF as one of the parameters. We plotted the cortical distance between electrode pairs against the distance between their RF centers to obtain MFs of 2.43 mm/° and 2.71 mm/° for the two monkeys, which were in good agreement with previous studies (Daniel and Whitteridge 1961; Dow et al. 1981; Xing et al. 2009). Based on these, the median cortical spread of LFP was 387 ± 20 μm for *Monkey 1* and 436 ± 20 μm for *Monkey 2*. These results are comparable with the findings of Katzner and colleagues (2009) and Xing and colleagues (2009).

Although the estimated spatial spreads for LFP were circumscribed, closer inspection of Fig. 3*A* revealed the presence of small fluctuations in the evoked responses that were far away from the RF center, especially for *Monkey 1*. These fluctuations were characterized by a positive peak at ∼100 ms after stimulus onset. Furthermore, these fluctuations were not symmetric about the center but instead, were more prominent for probes placed at azimuths farther from the vertical meridian. The reasons for these fluctuations, or their asymmetry, are unclear, but these are likely to be due to volume conduction, because they disappeared for CSD (Fig. 3*B*). These fluctuations did not affect our LFP RF size estimates, because we only considered RMS values between 40 and 100 ms. To account for the possible influence of these fluctuations in the LFP RF estimate, we repeated the analysis by taking RMS values between 0 and 200 ms. RF sizes were 0.25 ± 0.011° for *Monkey 1*, only slightly larger than the RFs obtained earlier. For *Monkey 2*, the RF sizes were similar (0.22 ± 0.003°). Thus the results did not critically depend on the choice of analysis window.

#### Estimation of LFP spread in the spectral domain.

**Fig. 5.**
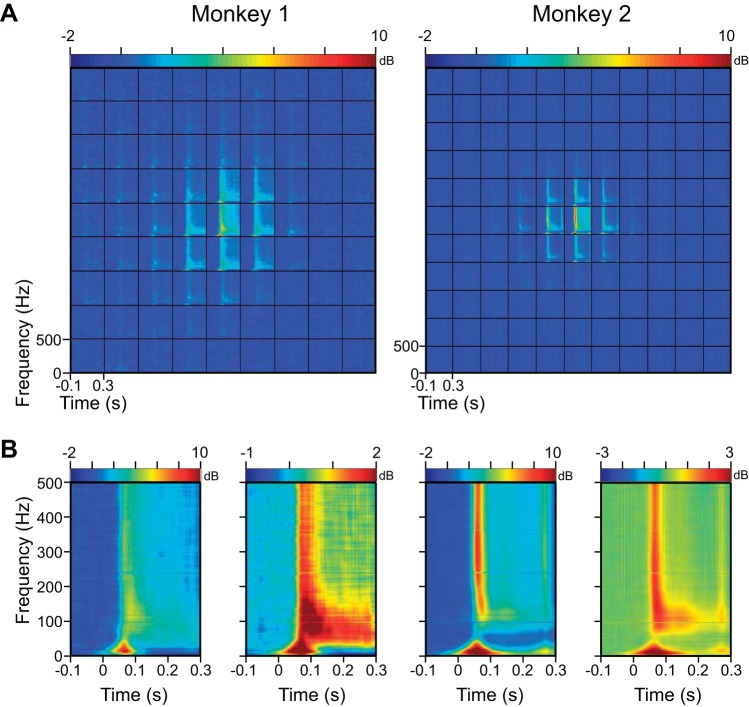
Time-frequency energy difference plots showing the change in energy [in decibels (dB)] from baseline (−100 to 0 ms; change computed separately at each frequency). *A*: time-frequency energy difference plots averaged across all electrodes (27 for *Monkey 1* and 71 for *Monkey 2*) after centering as in Fig 3. *B*: time-frequency energy difference plot at the central stimulus position (*left*) and the average across 4 stimulus positions diagonally 1 step away from the center (*right*). Note the difference in scale in these plots.

To study whether different frequencies of the LFP spread equally, the LFP signal was decomposed in the time-frequency domain using the MP algorithm (see materials and methods). Figure 5*A* shows the change in LFP power in decibels relative to baseline period (defined as −100 to 0 ms, where 0 corresponds with the stimulus onset; *Eq. 4*), centered and averaged across 27 and 71 electrodes for *Monkeys 1* and *2* (same procedure as in Fig. 3). At first glance, these time-frequency energy difference spectra also appeared circumscribed as the LFP. To observe the frequency dependence more clearly, we plotted the time-frequency difference spectrum for the center stimulus position and the average spectra of the four positions that were diagonally one step away from the center position (Fig. 5*B*^**^). At the center position, stimulus onset produced a strong transient broadband response between 40 and 100 ms, followed by an increase in power that was slightly higher in the high-gamma band but was also present at other frequencies. However, for the diagonal stimulus positions, the increase in power appeared more localized between 60 and 150 Hz.

**Fig. 6.**
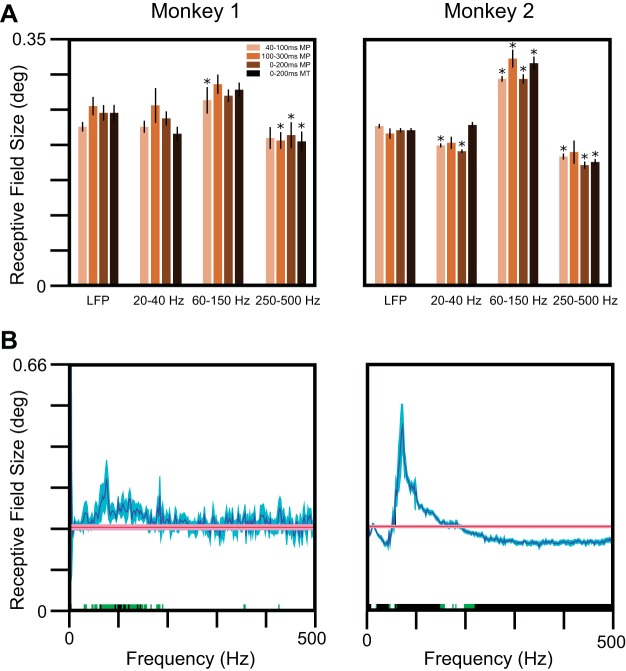
RF sizes in the spectral domain. *A*: median RF sizes across electrodes estimated using the LFP RMS values and using the total energy in 3 frequency bands (ranges indicated at the *bottom*), when the analysis interval was between 40 and 100, 100 and 300, and 0 and 200 ms after stimulus onset using matching pursuit (MP) and 0 and 200 ms using the multitaper (MT) method, as shown in the legend. The error bars indicate SE, obtained using bootstrapping. Frequency bands for which the RFs are significantly different from the LFP RF size are shown with asterisks (*P < 0.05, with Bonferroni correction for both multiple frequency ranges and time periods, Kruskal-Wallis test). *B*: median LFP spread across electrodes as a function of frequency (blue traces). For comparison, the RF size for the LFP is also shown (red traces). The SE, obtained using bootstrapping, is shown in lighter shades. Frequencies at which the medians are significantly different are shown in green (*P* < 0.05, without Bonferroni correction, Kruskal-Wallis test) or black (*P* < 0.05, with Bonferroni correction, Kruskal-Wallis test).

To test whether RF sizes varied with frequency, we estimated the RF sizes by fitting a 2D Gaussian (*Eq. 2*) to the difference in the root of the average energy values for stimulus (40–100 ms) and baseline (−100 to −40 ms) time epochs in three frequency bands (20–40, 60–150, and 250–500 Hz) and compared these with the RF computed without any filtering (same as LFP RF; Fig. 6*A*). The median RF size computed between 60 and 150 Hz was significantly greater than the RF size of LFP for both of the monkeys. In addition, we observed a reduction in RF size above 250 Hz (more prominent in *Monkey 2*), which is expected, as the high-frequency components of the LFP often reflect the firing rate of the neural population around the microelectrode (Manning et al. 2009; Ray et al. 2008a; Ray and Maunsell 2011a), and therefore, the RFs in this frequency range were similar to the RF sizes for the MUA.

Our results were similar when the energy was computed in the 100 to 300 or 0 to 200 ms range (Fig. 6*A*), although the increase in RF size was significant for *Monkey 2* only [the increase in RF size was observed for *Monkey 1* also, but the LFP RF was also higher in this duration due to the presence of a late positivity in the LFP trace, as described earlier (Fig. 3*A*), so the difference did not reach significance]. Importantly, for all of the time periods, the estimated RF size computed between 60 and 150 Hz was significantly greater than the RF size between 250 and 500 Hz for both of the monkeys (*Monkey 1*: *P* = 7.71 × 10^−5^, 4.62 × 10^−5^, and 6.07 × 10^−6^ for 40–100, 100–300, and 0–200 ms time periods; *Monkey 2*: *P* = 2.97 × 10^−21^, 2.13 × 10^−7^, and 5.55 × 10^−19^ for the same time periods).

The use of the MP algorithm allowed us to visualize better the frequency spread in the time-frequency domain (Fig. 5*B*), but the results were similar with other methods also. For example, we repeated the procedure after first computing the power using the multitaper method between 0 and 200 ms for the stimulus period and −200 to 0 ms for the baseline period (Fig. 6*A*; we used a single taper to achieve the best frequency resolution, and we could not use a shorter time window because the frequency resolution became very poor). The RF sizes for the raw LFP and for 20–40, 60–150, and 250–500 Hz frequency ranges were 0.24 ± 0.01°, 0.24 ± 0.01° (the median difference from LFP RF was not significantly different from 0; *P* = 0.48, *n* = 27, Kruskal-Wallis test), 0.27 ± 0.01° (*P* = 0.047), and 0.21 ± 0.02° (*P* = 0.004) for *Monkey 1* and 0.22 ± 0.003°, 0.23 ± 0.004° (*P* = 0.12, *n* = 71, Kruskal-Wallis test), 0.31 ± 0.01° (*P* = 3.25 × 10^−23^), and 0.18 ± 0.004° (*P* = 1.07 × 10^−10^) for *Monkey 2* compared with the results obtained using the MP algorithm. Thus our results did not depend on the choice of analysis technique.

We averaged the power in predefined frequency bands to make the estimate of power more reliable (Kajikawa and Schroeder 2011; Liu and Newsome 2006). To ensure that our results were not biased by the choice of frequency ranges, we repeated the analysis at each individual frequency as well (Fig. 6*B*). Although the results were noisier [for example, the reduction in RF size at higher frequencies (>250 Hz) was not observed for *Monkey 1*, potentially due to a smaller number of available electrodes], RF sizes showed a clear and significant increase between the 60- and 150-Hz range in both monkeys, reaching a maximum value of ∼0.35° and ∼0.49° at ∼75 Hz (not related to frame rate, which was set to 100 Hz), which corresponded to a cortical spread of ∼774 and ∼1,261 μm for the two monkeys. To test whether our results at higher frequencies did not depend on the choice of frequency range, we divided the 250- to 500-Hz band into three frequency bands (250–300, 300–400, and 400–500 Hz) and found that the estimated RF sizes for these bands were not significantly different from the RF size computed between 250 and 500 Hz (data not shown). This also shows that spatial spread did not have a low-pass filtering effect, which would have led to a progressively smaller RF size with increasing frequency.

#### Coherence analysis.

**Fig. 7.**
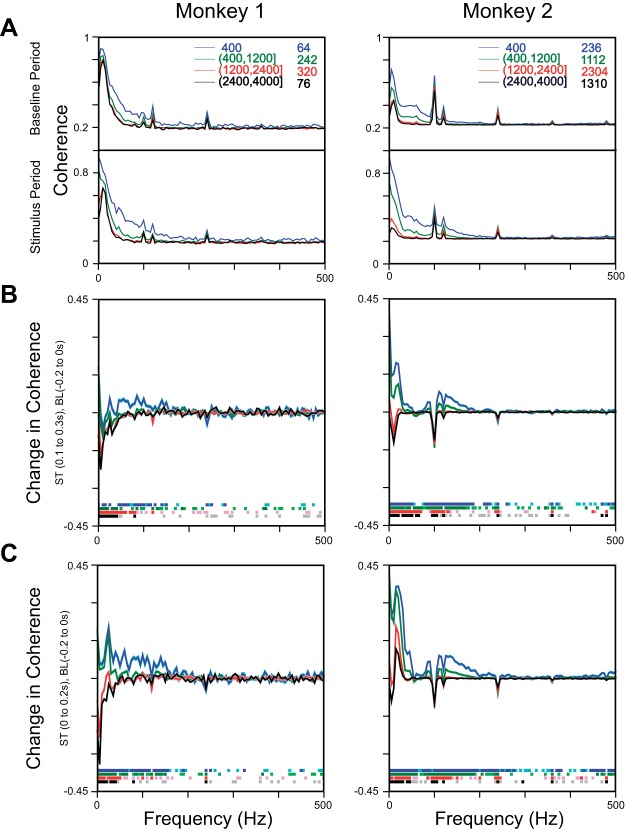
Phase-coherence analysis. *A*: median phase coherence across all electrode pairs for baseline (−200 to 0 ms) and stimulus (100–300 ms) periods, divided into 4 interelectrode distance ranges, as shown in the legend. *B*: difference in stimulus (ST) and baseline (BL) phase coherence for the 4 interelectrode distance ranges. The SE, obtained using bootstrapping, is shown in a similarly shaded color. Frequencies at which the difference was significantly different from 0 are shown in light (*P* < 0.05, without Bonferroni correction, Kruskal-Wallis test)- or dark (*P* < 0.05, with Bonferroni correction, Kruskal-Wallis test)-shaded color toward the *bottom*. *C*: same analysis as above but for baseline and stimulus periods of −200 to 0 ms and 0–200 ms, respectively.

The analysis described above shows the LFP spread to be band-pass, spreading significantly more in high-gamma range. In a recent study, Lindén and colleagues (2011) showed the dependence of spatial reach of LFP on correlated synaptic activity. To study whether this band-pass effect observed in the RF size was due to correlations in LFP signals, we computed the phase coherence between pairs of electrodes separated by different interelectrode distances (*Eq. 5*; see materials and methods for details). Figure 7*A* shows the median phase coherence across all of the electrode pairs for baseline (−200 to 0 ms) and stimulus period (100–300 ms) for four interelectrode distance ranges. The median difference in stimulus and baseline phase coherence (Fig. 7*B*) showed a clear increase in high-gamma range for small interelectrode distances (up to 400 μm in *Monkey 1* and 1,200 μm in *Monkey 2*). Figure 7*C* shows the same result when stimulus interval was chosen between 0 and 200 ms. Irrespective of the time epoch used for analysis, we observed an increase in coherence in the high-gamma range in both of the monkeys.

**Fig. 8.**
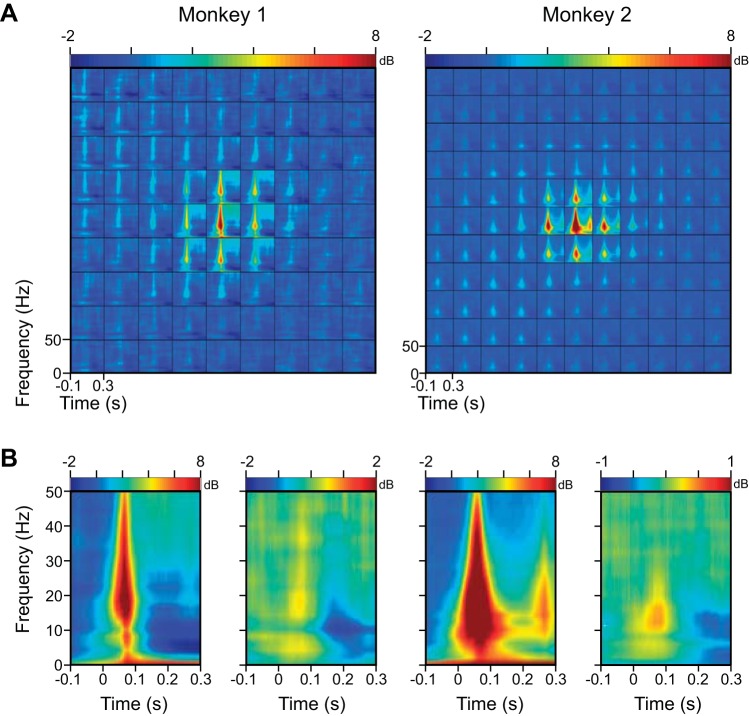
Time-frequency energy difference plots showing the change in energy (in decibels) from baseline (−100 to 0 ms; change computed separately at each frequency). *A*: same as Fig. 5*A*, but data are shown only up to 50 Hz to highlight the low-frequency components, as we observed a decrease in alpha band power (dark blue band). *B*: time-frequency energy difference plot at the central position (*left*) and the average across 4 extreme corner positions (*right*). Even when stimulus was far away from the RF center, there was a small onset transient and alpha suppression. Note the change in scale for the plot on the *right*.

Apart from the change in the high-gamma range, there was a prominent change at low frequencies, which was negative for *Monkey 1* but changed signs from positive to negative with increasing interelectrode distance for *Monkey 2* (Fig. 7*B*). This was because the power of the LFP at very low frequencies changed with stimulus presentation, even at stimulus positions that were far away from the RF center. Specifically, we observed a decrease in alpha band power (Fig. 8; which shows the same results as Fig. 5*A* but in the 0 to 50 Hz range). Alpha suppression was weaker in *Monkey 2* and was partially masked by a low-frequency transient at low frequencies (which was also observed at positions far away from the RF center). Therefore, stimulus onset suppressed alpha at all electrodes (whether or not stimulus fell inside of the RF of the electrode), which led to a reduction in the phase coherence in all electrode pairs. For nearby electrodes, however, stimulus onset also produced a low-frequency evoked response that contributed to an increase in phase coherence for short interelectrode distances. This effect was stronger in *Monkey 2* and also when the coherence was computed for the early stimulus period, during which the effect of transient activity was more prominent (Fig. 7, *C* vs. *B*). Thus changes in RF sizes were associated with corresponding changes in coherence between electrode pairs.

## DISCUSSION

We compared the spatial spreads of LFP, CSD, and MUA in the V1 and investigated whether the LFP spread was frequency dependent. The LFP spread was significantly larger than the CSD spread, which in turn, was significantly larger than the MUA spread, but all three spreads were fairly circumscribed, with the LFP spread in the range of ∼400 μm, consistent with previous studies of spatial spreads in the V1 (Katzner et al. 2009; Xing et al. 2009). As a function of frequency, the LFP spread varied in a band-pass manner, with a significantly larger spread in the high-gamma range compared with both lower (20–40 Hz) or higher (>250 Hz) frequencies. Spatial spread was also high for the alpha band but decreased to almost the spread of MUA at frequencies above 250 Hz. We found that the phase coherence across sites after stimulus presentation also increased in the high-gamma range, consistent with the findings of a recent model that shows that the spatial spread critically depends on neuronal correlations [interestingly, in their model also (Lindén et al. 2011), a small change in phase coherence (∼0.1) was enough to produce a large change in cortical spread].

Although our spatial-spread estimate was local, the results are not necessarily inconsistent with studies that showed a larger spread (Kajikawa and Schroeder 2011; Kreiman et al. 2006). In particular, we showed that a small stimulus presented far away from the RF of a particular electrode can nonetheless suppress the alpha rhythm or generate a transient low-frequency response recorded from that electrode (Fig. 8). Thus the spatial spreads can be large at some frequencies, which are also reflected in the phase-coherence profiles, and the differences in the spatial spreads reported in various studies could simply be due to differences in stimuli or cortical areas that could produce different levels of neuronal correlations (Lindén et al. 2011). The difference, however, could not be due to anesthesia [as suggested by Kajikawa and Schroeder (2011)], because our recordings were done on awake animals.

Although the main results were consistent across the two monkeys, there were many interindividual differences. These differences could potentially arise from differences in the depth at which the electrodes were inserted, which could be *layer 2/3* or *4* (see materials and methods). Horizontal connections made by the long, extending axon arbors, which are more prominent in superficial cortical layers (Fitzpatrick 1996; Gilbert and Wiesel 1983, 1989), may contribute to higher coherence and consequently a larger spatial spread in *layer 2/3*, which could be the case for *Monkey 2*. This is also consistent with the observation that the magnitude of gamma rhythm and LFP-LFP gamma-phase coherence, which are higher in the superficial layers (Buffalo et al. 2011; Xing et al. 2012), is considerably higher in *Monkey 2* compared with *Monkey 1* [Figs. 1, *F* vs. *I*, and 2*A* of Ray and Maunsell (2010)]. Intrinsic variability in the structural connectivity of the cortical network in the two monkeys or even in the microelectrode arrays could also have contributed to intersubject differences. Finally, changes in RF sizes could have failed to reach significance in *Monkey 1* due to fewer electrodes and recording sessions and consequently, lower statistical power [the upper half of the microarray grid was dysfunctional (Fig. 1), and the number of recording sessions was only 6 compared with 24 in *Monkey 2*].

### 

#### Previous studies on the frequency dependence of spatial spread.

The width of a somatic action potential recorded extracellularly has been shown to broaden with increasing distance from the soma (along with a reduction in amplitude) (Gold et al. 2006; Henze et al. 2000), which effectively implies a preferential suppression of higher frequency components. Several models have been proposed to explain this low-pass filtering effect (Bédard et al. 2004, 2006; Gold et al. 2006, 2007; Łęski et al. 2013; Lindén et al. 2010; Pettersen and Einevoll 2008; Reimann et al. 2013; Schomburg et al. 2012). For example, Bédard and colleagues (2004, 2006) explained this by assuming that the extracellular medium is capacitive, although this assumption is controversial (Bédard and Destexhe 2009; Bédard et al. 2010; Einevoll et al. 2013; Logothetis et al. 2007). Pettersen and Einevoll (2008) were able to show low-pass filtering even in a purely resistive medium, which was due to a shrinkage in the dipole length with increasing frequencies (which follows directly by solving the standard cable equation in the frequency domain). Specifically, potential was shown to decay as 1/r near the soma (where r is the distance from the point source) but as 1/r^2^ or faster once the “far-field” limit was reached, but this limit depended on the dipole length. At higher frequencies, this far-field limit was reached at a shorter distance, which led to a faster decay of potential with distance compared with the lower frequencies, yielding a low-pass effect. In subsequent studies, they showed a similar low-pass filtering of the LFP as well (Lindén et al. 2010) and described another factor—conversion of synaptic input correlations into higher correlations between single-neuron LFP contributions (measured using coherence) at low frequencies compared with higher frequencies, which also contributed to this low-pass effect [for details, see Łęski et al. (2013)]. In their simulations, effect of changing coherence on the spatial spread was much larger than the first factor related to shortening of the dipole length at higher frequencies.

In our data, although the coherence profile showed a low-pass shape during both baseline and stimulus epochs (Fig. 7*A*), coherence was actually close to zero at all frequencies beyond 800 μm if computed between CSDs instead of LFPs [see Fig. 4 of Srinath and Ray (2014)], which use data from the same monkeys; note that CSDs were computed by subtracting the average of the neighboring four electrodes spaced 400 μm apart, such that a proper estimate of coherence was possible only for electrodes spaced >800 μm [see materials and methods and Srinath and Ray (2014) for details], suggesting that the coherence was mainly due to volume conduction. Therefore, if coherence increased specifically in the high-gamma band after stimulus onset (for reasons that we speculate in the next section), then it would mask out the low-pass filtering effect due to dipole shortening and would also lead to a band-pass spatial-spread profile instead of low-pass filtering, as predicted by these models.

The difference in the coherence profile expected from the model (Łęski et al. 2013) and our data could arise from the differences in the assumptions used to define the spatial spread. We stimulated a small part of the cortex by presenting a small visual stimulus, but this activated group of neurons could also stimulate their neighboring neurons (through, for example, lateral connections). The spatial spread in this case is simply the overall area that gets stimulated when a small stimulus is presented. The modeling studies described above (Łęski et al. 2013; Lindén et al. 2010), on the other hand, used a passive circuit—an action potential in one neuron did not lead to more action potentials in the network (correlations in the population were generated by providing common inputs). It is possible that the subsequent activation of neighboring neurons from a centrally activated area could lead to a different coherence profile than the simultaneous activation of many neurons due to common input (as assumed in the model). Furthermore, because the model of Łęski and colleagues (2013) was passive, all of the power in the LFP was due to subthreshold processes; the model did not incorporate any low-frequency components associated with action potentials that can also contribute to the LFP, especially in the high-gamma range and above (Ray et al. 2008a; Ray and Maunsell 2011a; Schomburg et al. 2012). Addition of active elements in the model—in which generation of one action potential could generate more action potentials in the network, and these action potentials also could contribute to the LFP signal—could address some of these issues.

Although our results are inconsistent with model predictions as far as the spectral dependence is concerned, the absence of a low-pass filtering effect in our data is consistent with the findings of Kajikawa and Schroeder (2011), who measured the spatial spread in five frequency bands (1–2.9, 3–8.8, 9.1–26.7, 27.7–81, and 83.9–256 Hz) but did not find any reduction in the spread with increasing frequency. Their measurements, however, did not reveal any increase in the spatial spread in the high-gamma band either. This could be due to differences in the cortical area (auditory vs. visual) and stimuli (broadband noise stimuli vs. gratings) and also due to the spectral analysis method (wavelet vs. MP) and analysis interval (24 ms poststimulus onset vs. a longer interval in our study). Specifically, MP allowed us to generate time-frequency difference plots with high spectral and temporal resolution, which further allowed an appropriate choice of analysis interval and frequency range, although as discussed in results, once a suitable time interval was used, the results obtained using the multitaper method were similar (Fig. 6*A*). The similarity in the results between 0 and 200 ms using MP and the multitaper method (Fig. 6*A*) suggests that the onset-related transients and the violation of stationarity in this time interval did not severely affect the coherence analysis (for which typically, the first few hundred milliseconds are discarded, but here, this was not possible), since MP is a multiscale decomposition technique that has basis functions with narrow temporal support and is particularly well suited in representing stimulus onset-related transients [see Chandran KS et al. (2016) for details].

#### Other factors that can potentially influence the estimate of spatial spread.

Apart from the differences in the methods used for computing spatial spread (especially modeling vs. experimental approaches, as described above), there are some other factors that can influence the spatial spread. For example, it is unclear whether the results obtained at a particular eccentricity in the striate cortex can be generalized to other eccentricities. The dependence of the cortical point image (defined as the minimum cortical area activated by a point source in the visual field and approximately equal to the product of the MF and the population RF, or *MF*.*σ*_*vMUA*_, as defined in *Eq. 3*) on eccentricity has been studied extensively in V1, but the results are unclear. Some reports have shown that MF changes with eccentricity, according to a power law (Daniel and Whitteridge 1961; Dow et al. 1981; Van Essen et al. 1984), which makes the spatial spread greater in central vision than in peripheral vision, whereas others have reported a linear relationship between RF size and the inverse of the MF (Hubel and Wiesel 1974; Palmer et al. 2012), resulting in a constant spatial spread across eccentricity. Similarly, it is unclear whether the results can be generalized to the extrastriate cortex, where RF sizes are larger, or to regions of the brain that do not have an RF.

Another factor is the size of the visual stimuli used to compute the RF, which has been studied by Palmer and colleagues (2012). They showed that for stimuli much smaller than the cortical point image, the cortical spread is largely independent of stimulus size [see *Eq. 1* and Fig. 29.2*D* of Seidemann et al. (2009)]. In our experiment, we used Gabor patches of size 0.1° for *Monkey 1* and 0.05° for *Monkey 2* (see materials and methods for more details), and our results were comparable with Xing and colleagues (2009), who used sparse noise mapping to estimate the visual spread.

#### Origins of the coherence increase in the high-gamma range.

It is unclear why the coherence preferentially increased in the high-gamma band after stimulus onset. One reason could be simply based on the contribution of action potentials in the LFP, which have a visible contribution to frequencies as low as ∼20 Hz (Ray 2015; Ray et al. 2008a; Ray and Maunsell 2011a; Reimann et al. 2013; Schomburg et al. 2012). Synaptic inputs and low-frequency components of the action potential have power down to very low frequencies, but this contribution is masked out by the “1/f” noise in the LFP, which could be due to nonsynaptic contributions, independent of spiking activity (Buzsáki et al. 2012; Ray 2015), and could provide a high-pass effect. On the other hand, any jitter in the timing of occurrence of action potentials would affect higher frequencies more than low frequencies, since a constant jitter in time implies a larger change in phase angle at higher frequencies. This would generate a low-pass filtering effect. Together, high-gamma band in the LFP could be more sensitive to changes in spiking activity than lower as well as higher frequencies.

The possibility described above does not posit any special or functional role of the high-gamma band. However, several reports have hypothesized specific functional roles in oscillations at high frequencies, especially the classical gamma band (30–80 Hz), and our results could potentially be consistent with these proposals (Fries 2005, 2009). In previous studies, however, we have characterized gamma oscillations in these two monkeys (Ray and Maunsell 2010, 2011a; Srinath and Ray 2014) and found that they had peaks not exceeding ∼50 Hz, lower than the range described here. Furthermore, small stimuli do not produce substantial gamma (Gieselmann and Thiele 2008; Jia et al. 2013b; Ray and Maunsell 2011a). The use of a particular frequency band for preferential communication across brain areas is difficult at higher frequencies, because the power at higher frequencies is substantially less, and conduction delays or jitters in spike timings pose difficulties in maintaining a consistent phase (Ray and Maunsell 2015).

Whether the preferential spread of the LFP in the high-gamma range is used in the brain for interareal communication or information transfer would require additional experiments involving a cognitive task. Our results are useful in brain machine-interfacing applications (Andersen et al. 2014), since it is possible that power in the high-gamma band would be more robust toward recording positions (and also more selective to stimuli/tasks) (Berens et al. 2008; Liu and Newsome 2006) and also provide important clues about the cortical architecture, which can be used to build and improve large-scale models of the brain.

## GRANTS

Support for this work was provided by Wellcome Trust/Department of Biotechnology (DBT) India Alliance (500145/Z/09/Z; Intermediate Fellowship to S. Ray), Tata Trusts Grant, and DBT-IISc Partnership Programme.

## DISCLOSURES

No conflicts of interest, financial or otherwise, are declared by the authors.

## AUTHOR CONTRIBUTIONS

A.D. and S.R. conception and design of research; S.R. performed experiments; A.D. and S.R. analyzed data; A.D. and S.R. interpreted results of experiments; A.D. and S.R. prepared figures; A.D. and S.R. drafted manuscript; A.D. and S.R. edited and revised manuscript; A.D. and S.R. approved final version of manuscript.
